# A Systematic Scoping Review of the Current Applications of Digital Technology in Undergraduate Surgical Education

**DOI:** 10.7759/cureus.77278

**Published:** 2025-01-11

**Authors:** Fang Fang Quek, Stephen Meldrum, Jane Hislop

**Affiliations:** 1 Edinburgh Medical School, The University of Edinburgh, Edinburgh, GBR

**Keywords:** digital, education, learning, surgery, technology

## Abstract

Over the past few decades, technological advancements have established digital tools as an indispensable pedagogical resource in the realm of modern education. In the field of medical education, there is growing interest in how these digital tools can be effectively integrated to enhance undergraduate surgical education. However, despite their well-documented potential benefits, research specifically investigating the current use of digital technology in undergraduate surgical education remains limited, highlighting a critical gap in the existing literature.

To address this research gap, this scoping review aims to elucidate the current utilisation of digital technologies in undergraduate surgical education by addressing the research question: 'How are digital technologies currently being utilised in undergraduate surgical education to meet surgical learning outcomes'.

A scoping review was performed, adopting the Joanna Briggs Institute (JBI) framework. A comprehensive search strategy was conducted using the search terms 'technology' OR 'simulation' OR 'virtual reality' OR 'augmented reality' OR 'digital' OR 'online' AND 'undergraduate' AND 'surgical' on multiple electronic bibliographic databases including PubMed, Medline, ERIC, Embase, Scopus and Web of Science. These search terms were executed using both free-text and MeSH terms, with search terms combined using Boolean operators to ensure all relevant citations were captured. All search results were screened against the eligibility criteria using Covidence, a web-based software platform, using a two-stage process. Subsequently, all included studies were reviewed, and the extracted data was systematically sorted and organised, with the findings presented graphically accompanied by descriptive narratives. A thematic analysis was also performed to identify themes within the data to synthesise key findings.

This scoping review revealed three key findings. First, the use of digital tools in surgical education has been steadily increasing over the past few decades, with the COVID-19 pandemic accelerating the integration of technology into surgical education. Second, this review also highlighted the key role of anatomy within surgical education, with most included studies reporting the use of digital technologies to enhance anatomy teaching. Finally, this review provided an overview of various digital tools used in surgical education and their associated user experiences. Overall, most studies indicated that digital technologies are well-received by students, with many advocating for their continued use in supplementing surgical education even beyond the pandemic.

This review provides a crucial foundation for understanding the evolving role of digital innovations in shaping undergraduate surgical education. To enhance undergraduate surgical education, integrating appropriate digital learning tools can provide more learner-centred and personalised learning experiences. Educators must recognise that there is no ‘one-size-fits-all’ approach, and a flexible multimodal strategy is necessary to meet diverse learning needs. As technology continues to evolve and its role in education grows, this review offers valuable insights into the current use of digital tools in surgical education, highlighting opportunities for improvement and innovation to further enhance undergraduate surgical experience.

## Introduction and background

Undergraduate medical education is designed to prepare medical students with the knowledge and skills required to become competent doctors upon qualification, capable of delivering safe patient care [1]. In the United Kingdom, the majority of newly qualified doctors rotate through both medical and surgical specialties, making preparedness for surgical practice a key outcome of undergraduate medical education [2]. However, numerous studies have reported that a significant proportion of newly qualified doctors feel inadequately prepared for the transition to clinical practice [3,4]. In addition, the increasing demands on the healthcare system coupled with increasing clinical commitments have left surgeons with limited opportunities for teaching [5]. As highlighted by Lee et al., there is a pressing need for innovative approaches to surgical education to ensure that medical students achieve the essential surgical competencies within the constraints of modern clinical environments [6].

Digital technology, an umbrella term encompassing a wide range of electronic tools, systems and devices that generate, store and process data, has profoundly transformed many aspects of our lives and has gradually become an indispensable pedagogical tool in the realm of education [7-9]. Tools and innovations like the Internet of Things (IoT), 5G networks and artificial intelligence (AI) have revolutionised education [10]. Research has indicated that employing a variety of learning strategies, including the use of digital technology, is a crucial factor in determining academic success among undergraduate medical students [11,12]. As highlighted by Lochner et al., traditional didactic lectures, a commonly used approach in health sciences education, are often inadequate in effectively conveying large volumes of information as they do not provide the time needed for deep learning to happen [13]. Digital technology, on the other hand, offers the opportunity to transform these lectures by integrating them with online learning modalities to enrich the educational experience [14]. The integration of digital technology into medical and surgical education allows us to provide students with a more learner-centred and personalised learning experiences [12,15,16].

Therefore, one of the most important questions to be addressed is how digital technology can be effectively utilised to support teaching and learning in undergraduate surgical education. While much of the existing research on the use of digital technologies in medical education is focused on undergraduate medical education and in postgraduate surgical education, little research has been conducted to directly examine how technologies can be used to meet the surgical learning outcomes in undergraduate medical education. Studies directly examining the use of digital technologies in meeting undergraduate surgical learning outcomes are therefore few and far between. To answer the research question on how digital technology is currently utilised in the delivery of undergraduate surgical education, a systematic scoping review was conducted to explore its application in meeting surgical learning outcomes. A preliminary search of MEDLINE, the Cochrane Database of Systematic Reviews and JBI Evidence Synthesis was conducted and no current or underway systematic reviews or scoping reviews on this topic were identified.

Research question

This scoping review aims to assess and evaluate the scope of existing literature on the integration of digital technologies in undergraduate medical education, with a particular focus on their role in achieving surgical learning outcomes. For the purposes of this review, the term 'digital technology' is defined in accordance with Rice’s definition, encompassing a wide range of services, tools, applications and technologies that leverage software and hardware to generate, process or store data [17]. This scoping review also aims to identify literature gaps to inform future research.

## Review

Methodology and research design

Ethical Consideration

As this study involves working only with secondary data and does not involve direct interaction with human participants, formal ethical approval from ethics committees such as the Health Research Authority (HRA) is not required. In accordance with the BERA Ethical Guidelines, I hereby declare no conflict of interest and confirm that no funding was received for this study [18].

Research Design

This review seeks to address the research question: 'How are digital technologies currently being utilised in undergraduate surgical education?'. To address this research question, a scoping review was conducted in accordance with the Joanna Briggs Institute (JBI) methodology for scoping reviews, consisting of nine stages as outlined in Table 1 [19,20].

**Table 1:** Study methodology. Joanna Briggs Institute (JBI) framework for scoping review.

Stage 1	Identifying and aligning the objective(s) and research question. The objectives should relate directly to the review question and will describe what will be investigated/explored. This includes defining study population, interventions, and outcomes.
Stage 2	Developing eligibility criteria. Eligibility criteria should be directly linked to the research objectives and questions.
Stage 3	Describing the planned approach to evidence searching, selection, data extraction, and presentation of the evidence.
Stage 4	Searching for the evidence. Identify all relevant published and potentially unpublished evidence.
Stage 5	Selecting the evidence. Study selection based on pre-defined eligibility criteria.
Stage 6	Extracting the evidence. Using a standardised extraction form to extract data.
Stage 7	Analysis of the evidence. The intention of scoping reviews is to provide a map and summary of available evidence, not to synthesise results into a set of final findings to inform decision-making.
Stage 8	Presentation of the results.
Stage 9	Summarising the evidence.

Eligibility Criteria

Relevant studies in the literature are evaluated against the pre-defined eligibility criteria for inclusion. The established eligibility criteria for this scoping review are as follows.

Participants

The eligibility criteria for this scoping review focus exclusively on studies examining the current application of digital technologies within undergraduate surgical education, specifically among undergraduate medical students. Research involving participants from allied healthcare professions such as nursing, dentistry or other healthcare professions is excluded. This targeted approach ensures that this review concentrates solely on the unique educational needs of undergraduate medical students in achieving surgical learning outcomes. By narrowing the scope of this review to this population, this scoping review aims to provide precise and relevant insights into the integration of digital technologies within the context of undergraduate surgical training.

Concept

This scoping review encompasses all studies examining the use of digital technologies to support undergraduate surgical learning.

Context

Studies were included if they assess the use of digital technology within undergraduate medical education settings. Studies involving postgraduate medical and/or surgical trainees, defined as individuals who had already completed their training and qualified as doctors, were excluded. Additionally, only studies published in the English language were considered in this study, due to constraints on translation resources.

Search Strategy

As this is a scoping review, the aim of this study is to identify all relevant published and potentially unpublished evidence in the literature. Therefore, the search strategy must be sensitive enough to identify all relevant evidence but specific enough, so that the search does not capture an excessive volume of irrelevant results [20]. To achieve this, a comprehensive search strategy was conducted using the search terms 'technology' OR 'simulation' OR 'virtual reality' OR 'augmented reality' OR 'digital' OR 'online' AND 'undergraduate' AND 'surgical' on multiple electronic bibliographic databases including PubMed, Medline, ERIC, Embase, Scopus and Web of Science. These databases were selected for their high comprehensiveness in relation to the specific research question at hand.

During the preliminary search for studies pertinent to this research question, terms and phrases extracted from the titles, abstracts and indices of relevant papers guided the formulation of the search strategy. To broaden the scope of the search, grey literature was also utilised using internet search tools such as Google Scholar to identify informative sources. These terms were then refined, and all relevant search terms were identified to readjust the search strategy by consultation with an external expert colleague, an experienced research librarian from the University of Edinburgh. To ensure that all relevant records would be retrieved regardless of the terminologies used by the author, searches were executed using both free-text and MeSH (Medical Subject Headings) terms. The search terms were also combined using Boolean operators to ensure all relevant citations were captured.

A pilot trial of the initial search strategy revealed that the search terms 'undergraduate' and 'surgical' had to be included to limit the scope to undergraduate medical students and the use of digital technology in surgical education. To identify all pertinent studies on this subject and to explore if there was any change in the trend of technology use in the delivery of undergraduate surgical teaching, a start date for the search was not imposed. The last search was performed on January 5th, 2024.

Study Selection

This review was conducted in accordance with the Preferred Reporting Items for Systematic Reviews and Meta-Analyses (PRISMA) guidelines to ensure methodological rigor, transparency and reproducibility. All potentially relevant results yielded from the search strategy described were assessed for relevance based on the predefined eligibility criteria. This was performed in two stages. In the first stage, the titles and abstracts of all retrieved results were screened for potential inclusion. In the second stage, all studies deemed eligible were subjected to full-text screening and further assessed against the pre-defined eligibility criteria. To enhance the efficiency of the review process, Covidence, a web-based software platform, was utilised in the screening process.

**Figure 1:**
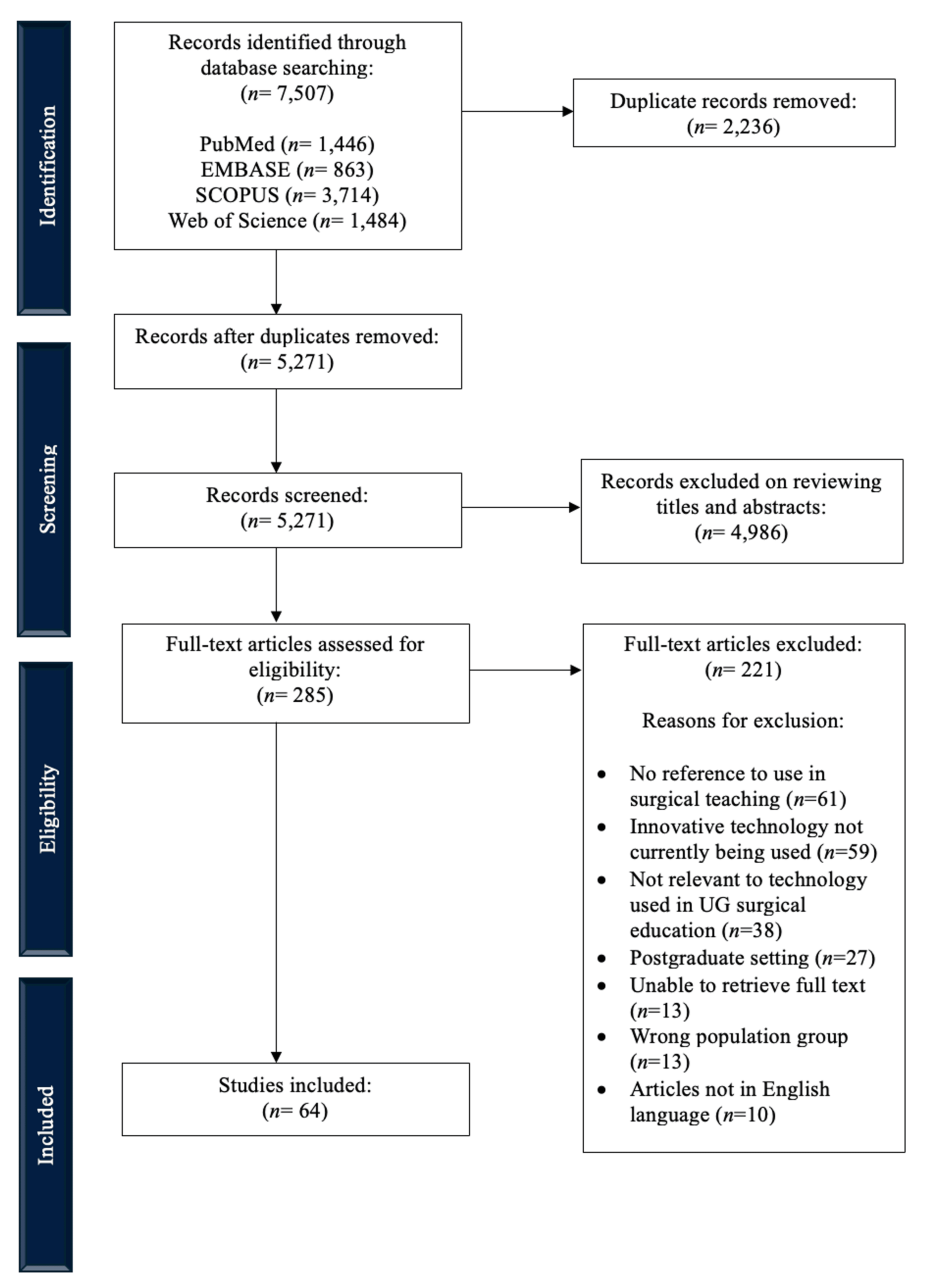
PRISMA flow diagram for the study selection process. PRISMA, Preferred Reporting Items for Systematic Reviews and Meta-Analyses.

A total of 7,507 articles were initially identified through the search process. Following the removal of duplicate studies (n = 2,236), the first screening stage led to an exclusion of 4,986 studies, leaving 285 articles for full-text screening. Subsequently, 64 studies met the eligibility criteria and were included for the final analysis. See Figure 1 for a diagrammatical representation of the study selection process.

All included studies were meticulously reviewed and data were systematically extracted using a standardised data extraction form created in Microsoft Excel. A charting form was created to record the essential characteristics and key information from each study. This data extraction form was pilot-tested to ensure its effectiveness and was refined based on the initial findings. Table 2 illustrates the data capture form utilised in this scoping review.

**Table 2:** Data capture form for this scoping review.

Title
Author(s)
Journal
Year of publication
Country of study
Format of paper (e.g., paper, abstract, letter)
Type of work (e.g., original research, review, opinion)
Number of participants
Year of study of participants (which year in undergraduate medicine)
Area/specialty where technology is used
Goal of technology use (e.g., for teaching or to assess competency)
Relation to COVID-19 pandemic
Aims of study
Methodology
Key findings
Conclusions
Reported challenges and/or limitations
Recommendations
Coding notes for thematic review

All collected data were sorted and organised with the aim of presenting them graphically with a descriptive narrative. Then, a thematic analysis of the included studies was performed to identify, analyse and report common themes within the data to allow for conclusions to be drawn based on common patterns identified across the included studies. To visualise the thematic connections, a thematic 'tree' was constructed, illustrating the relationships between the identified themes. This structured approach ensured a robust and comprehensive synthesis of the data, offering insights into the relationships underpinning the findings. 

Presentation of findings

Types of Publications

**Figure 2:**
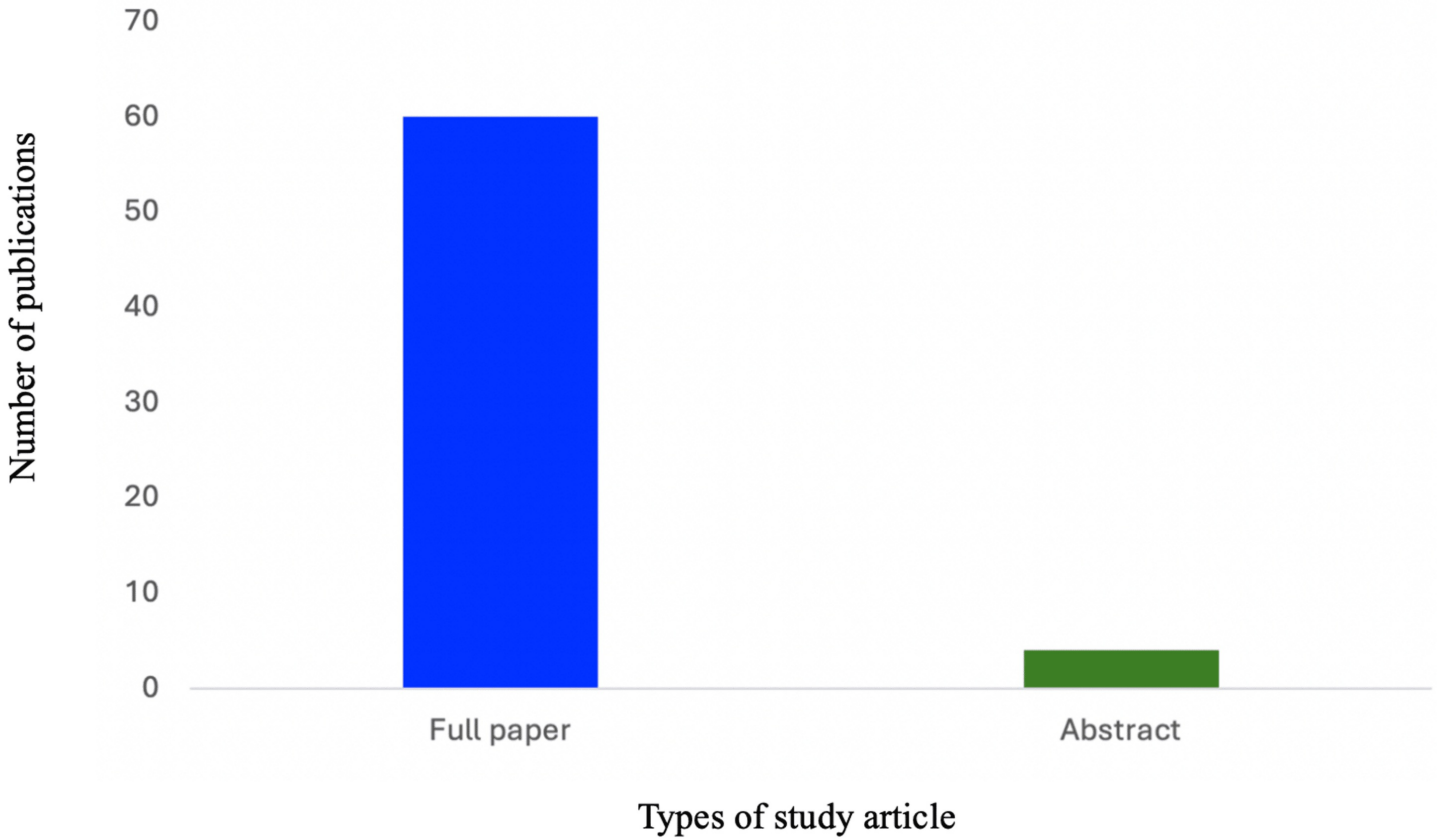
Types of included studies.

Of the 64 studies included in our study, 60 were published as full papers and four were published as abstracts. As illustrated in Figure 2, the majority of the included studies were original research (n = 58), with five review papers and one opinion paper.

Years of Publications

**Figure 3:**
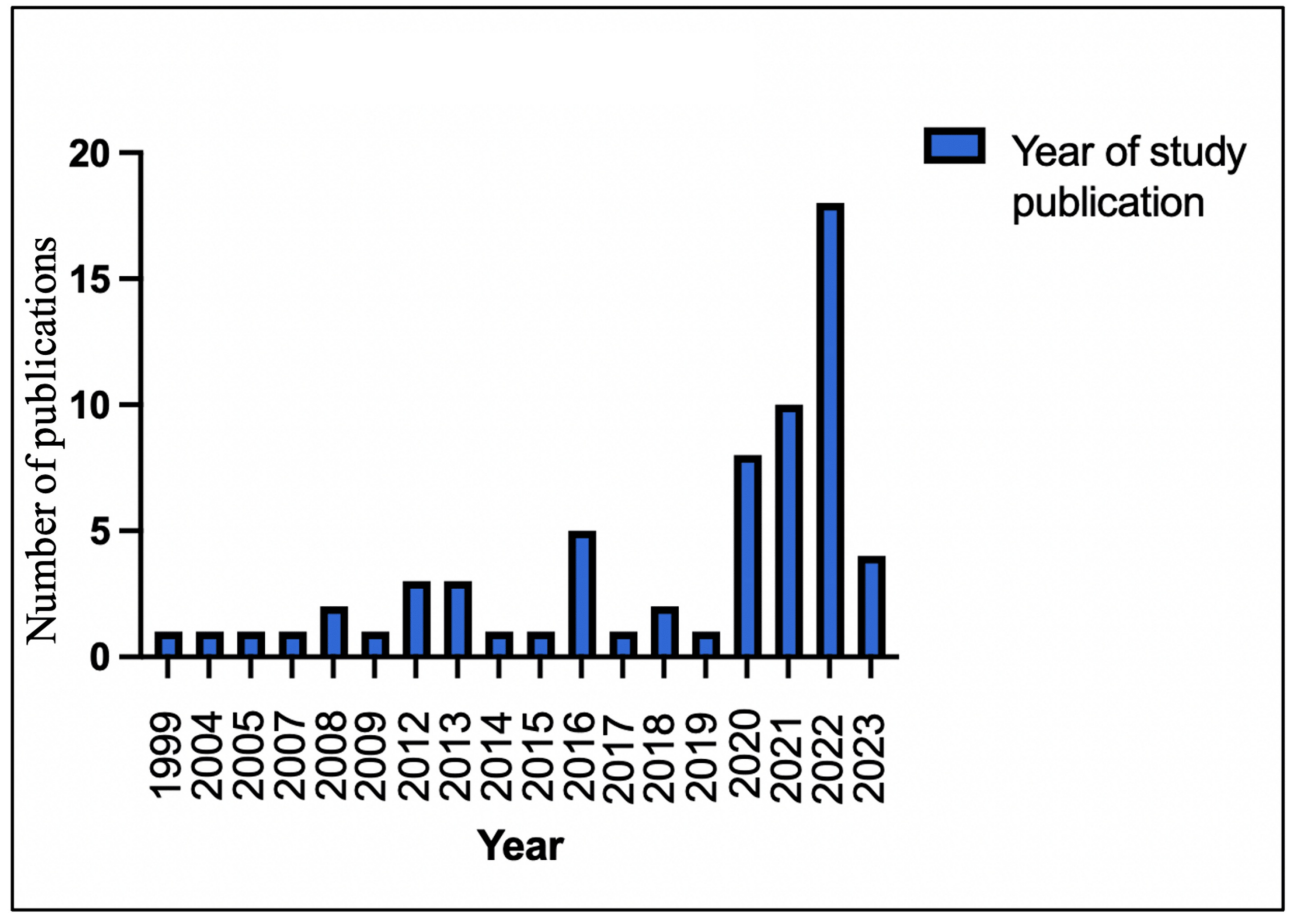
Years of publications of included studies.

To capture all relevant studies on this research topic and to assess if there was any change in the trend on the utilisation of technology in the delivery of undergraduate surgical teaching, a specific date range was not imposed for the search. The papers included in this scoping review were published between 1999 and 2023. Among the included articles, the earliest study was published in 1999. A notable increase was observed in the number of published papers, particularly from 2020, with 40 out of the 64 included papers being published during this period, suggesting a recent increase in interest in this field, likely related to the emergence of the COVID-19 pandemic as discussed in the following section. Figure 3 illustrates the publication timeline of the studies included in this scoping review.

Studies Related to COVID-19 Pandemic

**Figure 4:**
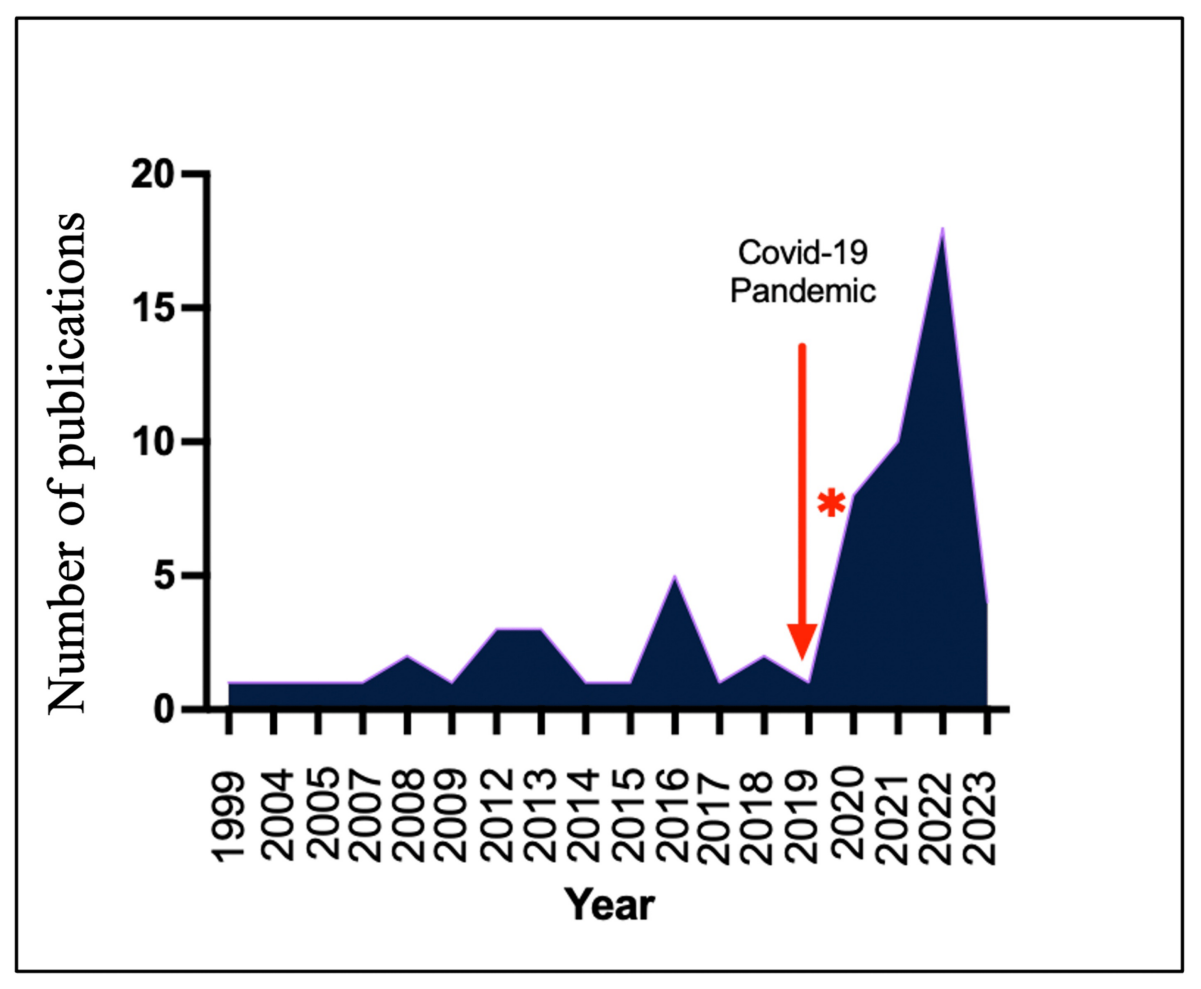
Number of publications according to years. *Graph showing a significant increase in the number of publications on the utilisation of digital technology in the delivery of undergraduate surgical education since the COVID-19 pandemic.

**Figure 5:**
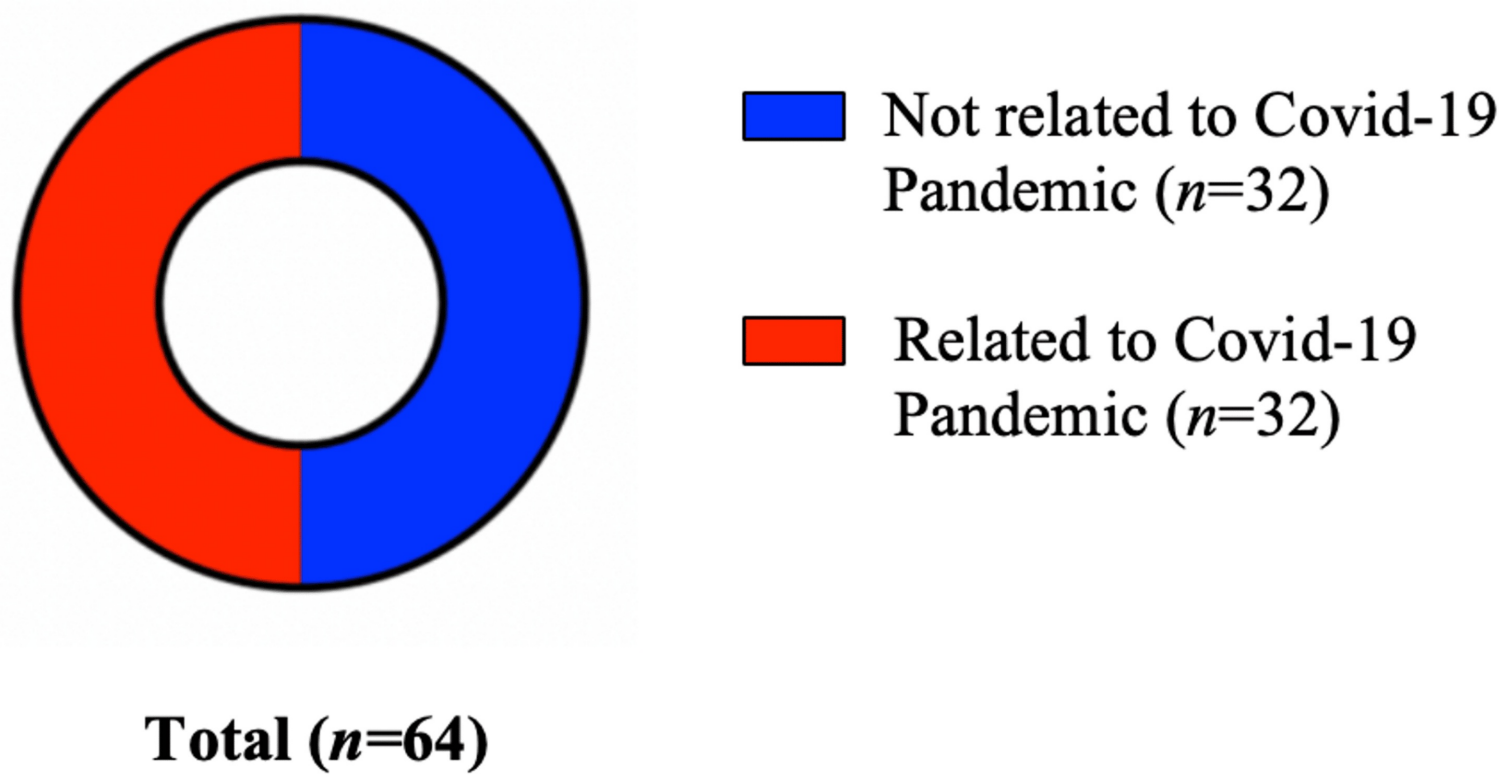
Studies related to COVID-19 pandemic.

Figures 4, 5 illustrate the notable surge in the number of publications on the utilisation of digital technology in the delivery of undergraduate surgical education following the COVID-19 pandemic. Out of the 64 studies included in this scoping review, a significant proportion (32 out of 64 (50%)) of studies specifically addressed the impact of the COVID-19 pandemic on technology use in the delivery of undergraduate surgical education. Additionally, 17 studies specifically referenced the COVID-19 pandemic in their titles. This finding highlights a significant shift towards the utilisation of digital tools and platforms in undergraduate surgical education since the COVID-19 pandemic.

Geographical Locations of Studies

**Figure 6:**
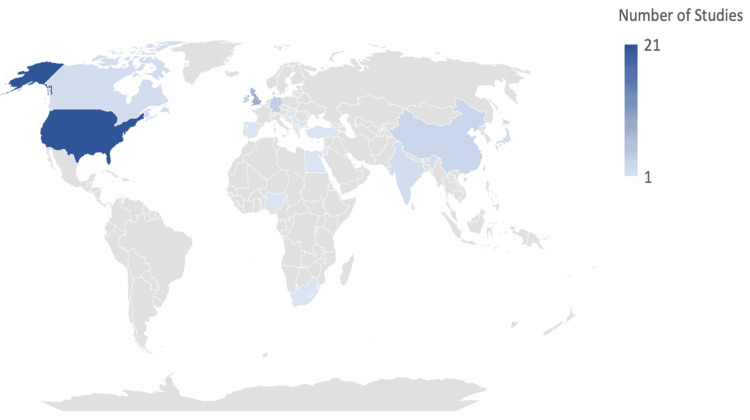
Geographical distribution of studies included in the review.

Most studies included in this review were conducted in the United States (21 out of 64), followed by a notable number from the United Kingdom. The remaining studies were distributed across various countries, as illustrated in Figure 6. Altogether, the studies included in this study originated from 22 different countries, highlighting a diverse geographic representation.

Integration of Digital Technology in Undergraduate Surgical Education Across Specialties

**Figure 7:**
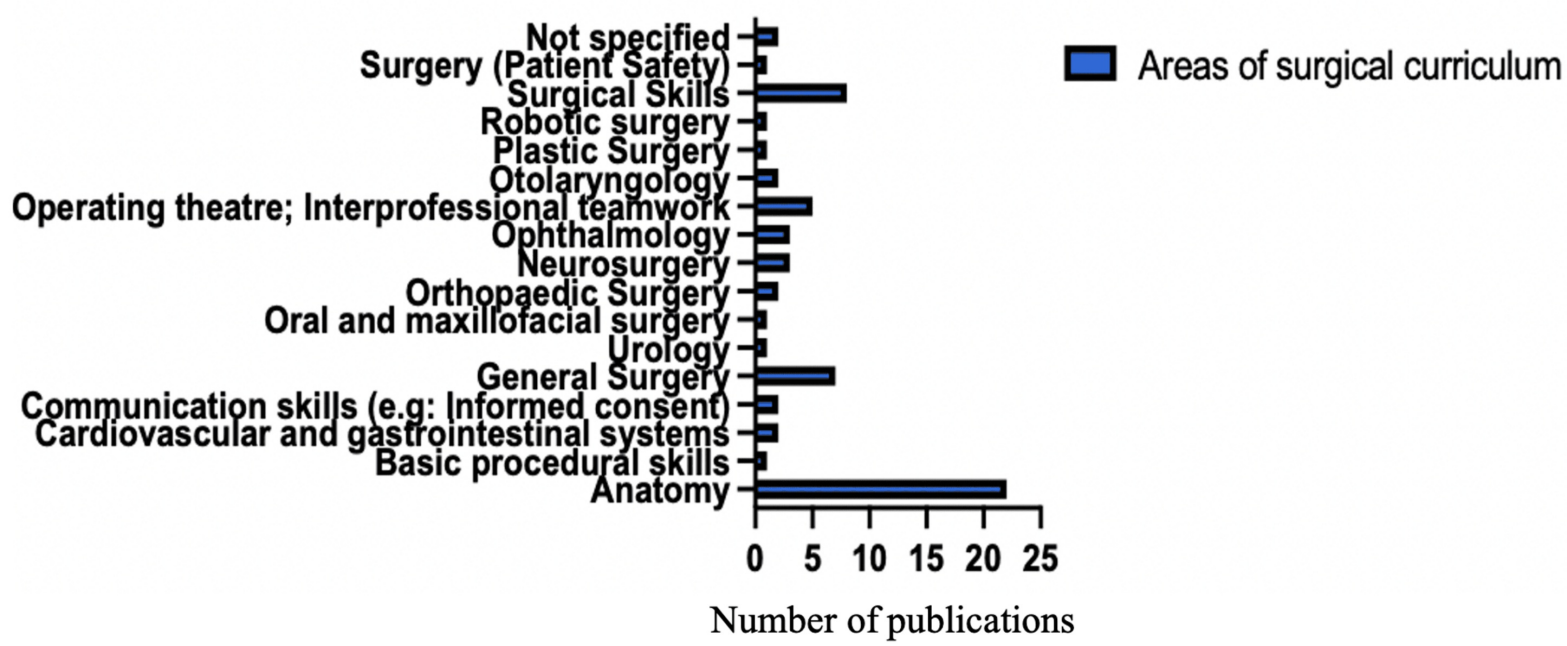
Areas of surgical curriculum where technology is used.

As shown in Figure 7, digital technology has been implemented across a range of surgical specialties. Of the 64 studies included in this scoping review, the largest proportion (22 studies) focused on the application of digital technology in teaching anatomy. This was followed by studies examining its application in teaching general surgery and surgical skills to undergraduate medical students. The remaining studies explored the use of digital technology across various other surgical specialties, as detailed in Figure 7, highlighting its broad and versatile integration into undergraduate surgical education.

Objectives of Digital Technology Utilisation in Undergraduate Surgical Education

**Figure 8:**
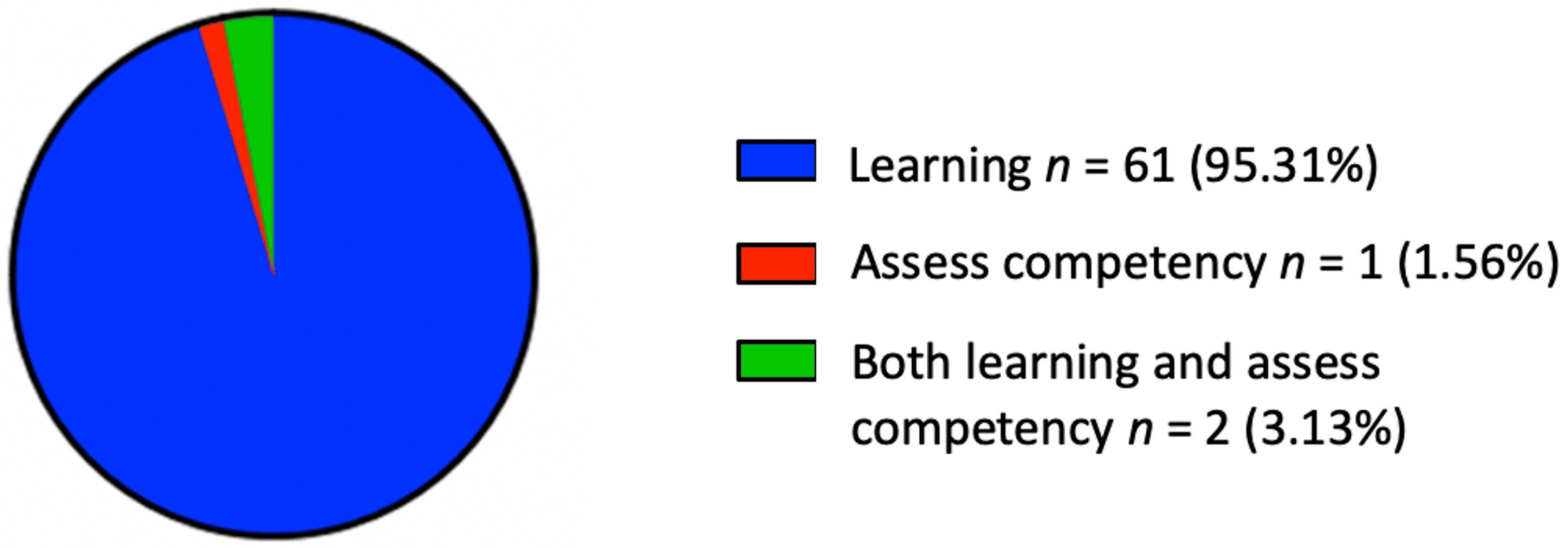
Reported objectives for the use of digital technology in undergraduate surgical education.

Figure 8 highlights the primary objectives for integrating digital technology into undergraduate surgical education, as identified in the reviewed studies. Of the 64 studies included, 61 primarily focused on the utilisation of digital technology to enhance teaching, emphasising its role in facilitating educational delivery. In contrast, only one study centred exclusively on using digital technology for the assessment of students’ competencies. The remaining two studies explored a dual approach, examining the application of digital technology for both teaching and assessing purposes.

Types of Digital Technology Currently Used in Undergraduate Surgical Education

**Figure 9:**
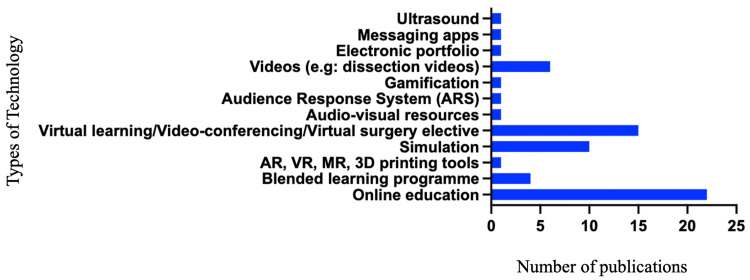
Breakdown of the technologies used in the included articles.

A wide range of digital technologies has been employed to support and enhance undergraduate surgical education. Figure 9 illustrates the diverse types of technology utilised to achieve surgical learning outcomes. An analysis of the 64 included studies revealed that computer-assisted learning (CAL) was the most prevalent pedagogical approach, cited in 51 studies. This encompasses online education, multimedia tools such as videos, blended learning, flipped classrooms and advanced technologies like virtual and augmented reality. Notably, online education or eLearning emerged as the most frequently reported modality, with 22 studies reporting its use in delivering educational content. Six studies specifically described the utilisation of dissection videos for anatomical teaching, while four studies highlighted blended learning programmes that integrate online and traditional classroom-based lectures.

Simulation-based learning (SBL) was described in 10 studies, and virtual learning environments were reported in 15 studies. The remaining studies reported the utilisation of a variety of technologies, including audio-visual resources, electronic portfolios, messaging applications, ultrasound tools, and gamification, demonstrating the breadth of digital technologies as pedagogical approaches in undergraduate surgical education.

While there are numerous studies in the literature reporting on the potential use of cutting-edge technologies such as virtual reality, augmented reality, mixed reality, and 3D printing tools in surgical education, only one study reported on the current application of these advanced tools in undergraduate surgical education.

Trends in Digital Technology Usage Across Undergraduate Medical Years

**Figure 10:**
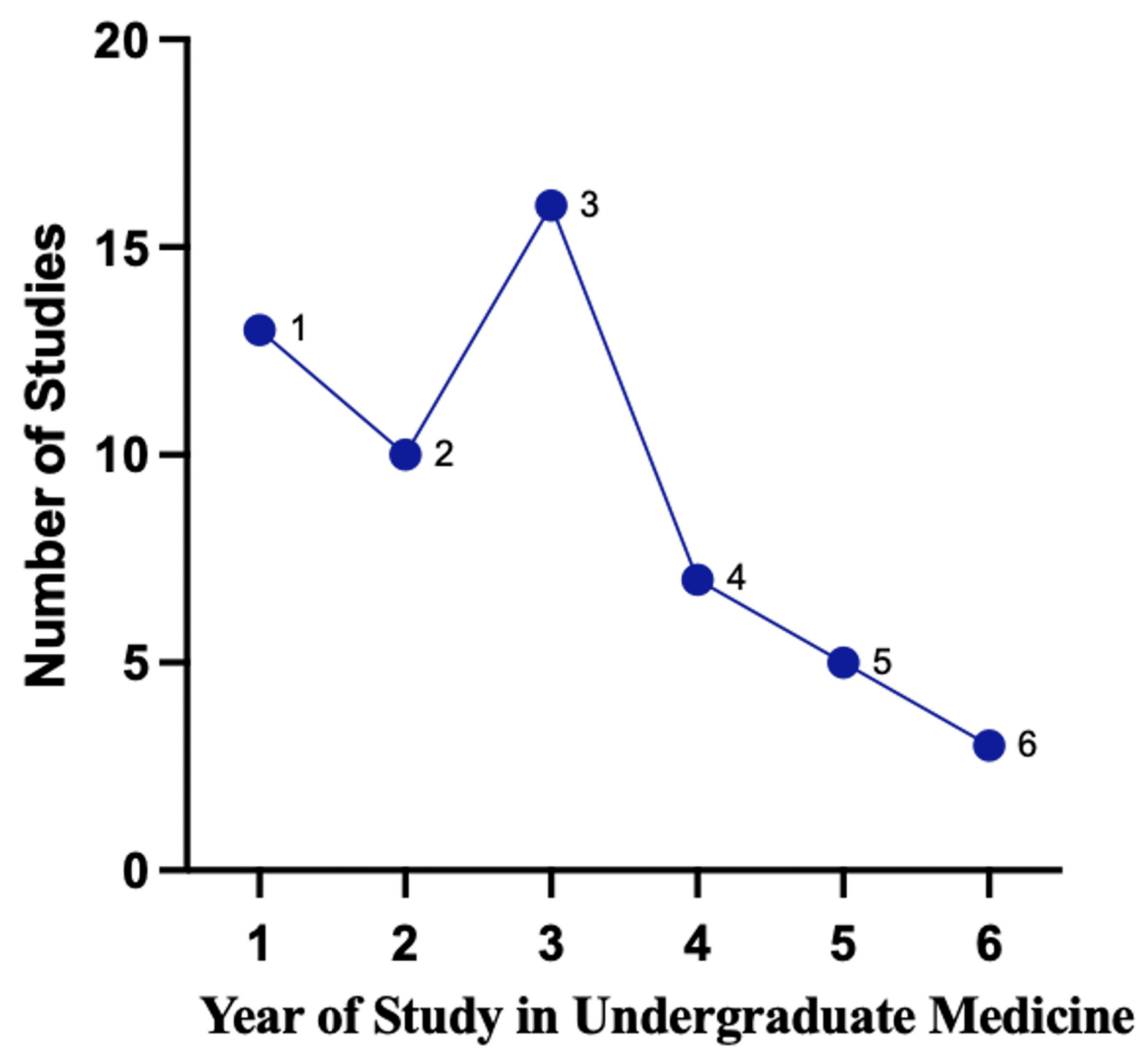
Number of studies reporting the utilisation of digital technology across different years of study.

Of the 64 studies reviewed, 10 did not specify the year of undergraduate medical study in which the discussed digital technology was employed. Among the remaining 54 studies, a substantial majority (39 studies) reported the use of digital technology during the pre-clinical years (Years 1-2 or Years 1-3, depending on the institutional structure) to support undergraduate surgical education to meet surgical learning outcomes. However, there was a notable decline in the reported use of digital technology as students progressed to later years of study, highlighting a potential gap in its integration into clinical training during advanced stages of medical education. Figure 10 shows the distribution of digital technology usage for surgical education across different years of undergraduate medical study.

Thematic analysis

Codes identified from the thematic analysis were subsequently grouped into three overarching themes relating to the utilisation of digital technology in delivering undergraduate surgical education to meet surgical learning outcomes. Following the thematic analysis, the following three themes were identified:

1. The acceleration of the integration of digital technology into surgical education following the COVID-19 pandemic

2. Integration of digital technology in undergraduate surgical education across specialties

3. User experience.

**Figure 11:**
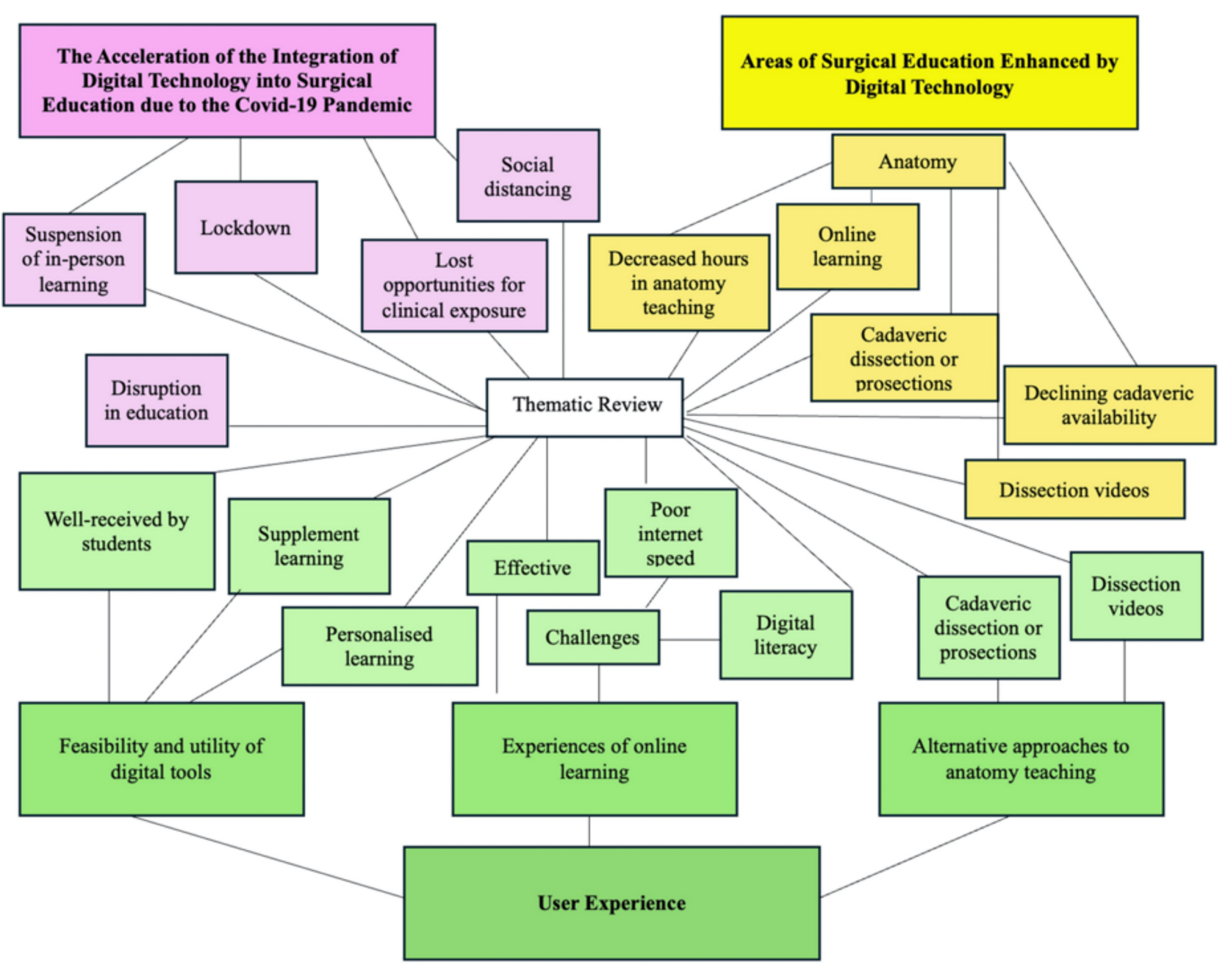
Diagrammatic representation of grouping decisions for thematic review.

Figure 11 shows the diagrammatic representation of the grouping decisions for this thematic review. 

Discussion

The Acceleration of Digital Technology Integration Into Surgical Education Following the COVID-19 Pandemic

The adoption of digital technology in medical education has been steadily advancing over the past decades, but its implementation surged dramatically with the onset of the COVID-19 pandemic [21]. Among the studies included in this review, 50% explicitly addressed the impact of the COVID-19 pandemic on the integration of digital technology into undergraduate surgical teaching. Notably, 17 studies mentioned COVID-19 in their titles, emphasizing its transformative impact. For example, Boulos’ paper, 'Evaluation of the Effectiveness of Online Education in Anatomy for Medical Students During the COVID-19 pandemic', begins by describing the pandemic’s substantial disruptions to educational systems [22]. Similarly, Dedeilia et al., in their study titled ‘Medical and Surgical Education Challenges and Innovations in the COVID-19 Era: A Systematic Review’, highlight the pandemic’s profound effects on surgical education [1]. Across the reviewed studies, the COVID-19 pandemic consistently emerged as the most frequently cited catalyst for digital transformation, with digital technologies playing a crucial role in bridging the educational gaps during this pandemic [23].

The unprecedented emergence of COVID-19 in late December 2019 marked a global health crisis, characterised by its rapid and widespread dissemination across the globe [24,25]. The pandemic has resulted in a significant loss of human life globally and has caused widespread disruptions across all facets of life, including our educational systems [22]. The closure of academic institutions as part of the containment measures to curb the spread of the disease, alongside the reallocation of academic medical trainees to frontline clinical roles, significantly strained the medical education systems [26,27]. UNESCO (United Nations Educational, Scientific and Cultural Organisation) reported that more than 900 million learners at all educational levels were affected by these disruptions globally [26,28]. Medical students, in particular, faced restrictions on clinical rotations and lectures due to safety concerns and efforts to mitigate the risk of transmission [29,30]. Surgical education, in particular, faced even greater challenges during this pandemic. The cancellation or postponement of elective surgical procedures and clinics, alongside the shift of educational conferences to virtual formats, severely reduced the learning opportunities for students [31]. To address these challenges and minimise educational disruptions, digital technologies were increasingly introduced or repurposed to facilitate surgical education, maintaining educational continuity during unprecedented crises [23,26].

Integration of Digital Technology in Undergraduate Surgical Education Across Specialties

The analysis of all studies included in this scoping review reveals a predominant focus on anatomy education, with 22 of the 64 studies reporting the integration of digital technology in this domain. This finding aligns with the universally acknowledged importance of anatomy as a cornerstone of modern clinical practice [32,33].

A recurrent theme across these studies is the pivotal role of anatomical knowledge in clinical practice and the innovative ways digital technologies have been utilised to enhance anatomy teaching [34,35]. For example, Abdullah et al. described anatomy as the 'cornerstone of healthcare education', emphasising the need to integrate resources to enhance anatomy learning [36]. Similarly, Adnan and Xiao referred to anatomy as 'the prerequisite for medical education', and further described how technology has been used to support anatomy learning [37]. With the reduction in resources allocated for anatomy teaching, including the allocated time, staffing and cadaveric availability, the pressing need for alternative approaches to anatomy teaching has been widely recognised [36,38-40]. While this review highlights the prominence of anatomy teaching in the adoption of digital technologies, a more detailed discussion of these findings and their broader implications will be explored in a subsequent publication.

Types of Digital Technology Currently Utilised in the Undergraduate Surgical Education

Computer-assisted learning: CAL is the most widely reported technology used in delivering undergraduate surgical education across the included studies. With the advancement in technology and the widespread adoption of CAL, it has become an integral tool in modern education, offering flexibility and personalised learning experiences [36,41,42]. CAL is defined as any educational experience facilitated through a computer interface and encompasses a variety of resources, including electronic/online learning (e-learning) platforms, 3D software, video materials, and virtual and augmented reality [36,43-45]. Studies have reported the effectiveness of CAL in supplementing traditional learning methods, enhancing students' academic performance and improving their achievements [46]. Commonly cited advantages of CAL include its provision of asynchronous learning, which allows students to control the pace of their studies, thereby fostering learner independence and accommodating diverse learning needs [36,47,48].

An essential competency expected of medical students is the ability to translate theoretical knowledge into clinical practice [49]. To support this, medical schools worldwide have increasingly adopted case-based (CBL) or problem-based learning (PBL) as modern pedagogical approaches which promote active learning, moving away from traditional rote memorisation and passive learning methods [50-52]. CBL encourages self-directed learning through active engagement and participation [53,54]. However, implementing CBL in traditional lecture formats can be challenging due to the difficulty of presenting a diverse range of clinical cases [49]. Additionally, large-group CBL sessions often result in low student participation and interaction, which can lead to diminishing motivation and learning outcomes [51]. To address these challenges, technological tools like CAL have been employed to present a wider variety of cases through online platforms, thereby enhancing self-directed learning and strengthening problem-solving skills [55]. 

Audio-response system (ARS): One study including this review examined the use of an ARS, commonly known as ‘clickers’, in educational settings, demonstrating its ability to enhance and maintain motivation among both students and instructors [56]. ARS consists of small, hand-held devices that enable students to respond instantly to questions posted during lectures, with responses instantly tallied and displayed, thereby providing real-time feedback for both students and instructors [56,57]. Consistent with previous research, the study found that ARS effectively facilitates CBL, serving as an effective tool to promote active engagement and participation among students [58]. By encouraging interaction and providing immediate feedback, ARS helps to maintain student interest and involvement, further enhancing the overall learning experience.

Electronic/online learning (e-learning): This scoping review identified 20 studies reporting on the adoption of e-learning in surgical education. With the continuous advancement in educational technologies, e-learning has evolved from a passing trend to a mainstream pedagogy in medical education [59-61]. E-learning is defined as the use of electronic technology to deliver and enhance learning to learners, often at remote locations, facilitating communication between instructors and students through online content [59,62]. In response to the increasing pedagogical challenges, such as the increasing clinical demands on clinical tutors, there has been a paradigm shift towards online learning in UK medical schools [63]. During the COVID-19 pandemic, e-learning emerged as the most widely accepted approach, ensuring the continuity of education despite disruptions [22,64].

Learning is inherently a personal experience and research has consistently shown that learner engagement is the most critical factor in determining success in higher education [65,66]. The use of multimedia elements such as animation, audio and video in e-learning has been found to significantly enhance learners’ engagement by fostering increased participation, which in turn promotes deeper cognitive engagement [67,68]. One of the studies identified in this scoping review discussed the use of the flipped classroom model as a pedagogical approach in e-learning [69]. The flipped classroom represents a transformative teaching strategy where students first engage with course material online through self-directed learning, allowing them to develop a foundational understanding before attending in-person classes [69,70]. It has been shown that this approach not only increases student motivation but also promotes active learning [71,72].

E-learning can be delivered through either synchronous or asynchronous methods [73]. Synchronous learning involves real-time interaction through technologies such as teleconferencing and instant messaging, closely resembling traditional classroom instruction [74]. In contrast, asynchronous learning refers to learning where the transmission and receipt of information do not happen in real time, allowing learners to engage with course materials at their own pace, offering greater flexibility and supporting personalised learning [68,75,76].

Videoconferencing: Another frequently cited technology in the delivery of surgical education across the studies in this scoping review was videoconferencing and virtual electives. Videoconferencing is a technology that allows live, real-time broadcasts between participants, exemplifying synchronous online learning [77, 78]. One of the key advantages of videoconferencing is its ability to deliver education remotely, which is particularly beneficial for students located in remote areas, thereby enhancing equitable access for those completing rotations in distant hospitals [77]. Many studies in the literature have noted that these tutorials are often supplemented with self-directed learning materials, such as videos, presentations and quizzes, to enhance the overall educational experience [30,79]. Research has shown that videoconference lectures can convey an equivalent amount of knowledge as traditional face-to-face lectures [80]. However, challenges such as the logistical coordination required for students across multiple locations and the need for adequate telecommunication equipment can impede the smooth implementation of videoconferencing tutorials [77]. Therefore, effective planning, interactive teaching strategies and dedicated coordinators are essential for the successful facilitation of these sessions [77].

Furthermore, two studies in this scoping review reported on the experiences of students participating in virtual surgical electives [27,79]. The increasing workload and resource constraints within the healthcare system have led to a notable shift of educational responsibilities from teaching faculty to specialty trainees [81]. As a result, medical students often find themselves relegated to passive observational roles in operating theatres, which limits their active participation in surgical procedures [82]. These virtual surgical experiences emerged as a valuable tool, providing students with an interactive way to continue their surgical education, even in situations where hands-on involvement is not feasible [30].

Blended learning: Blended learning has emerged as an innovative approach in undergraduate surgical education, as documented by several studies included in this review [69,83]. This method combines the strengths of e-learning and traditional classroom instruction, creating a more dynamic and flexible educational environment. For example, Funke et al. reported the use of a web-based, interactive e-learning platform, designed as a virtual hospital, to supplement surgical teaching for undergraduates [83]. Research has shown that blended learning not only enhances knowledge acquisition, it also fosters self-directed and metacognitive skills, as students are encouraged to take greater responsibility for their learning [84]. Additionally, the face-to-face elements of blended learning address the limitations of online education by facilitating meaningful student-tutor interaction and promoting collaborative learning [85].

Messaging apps: One study included in this scoping review investigated the use of messaging apps in surgical education [21]. Social media platforms, now deeply embedded in our everyday lives, are increasingly recognised for their potential to revolutionise continuing medical education [86,87]. Social networking sites (SNS) which include blogs, networking platforms, content-sharing sites and messaging apps are online spaces where users create profiles, connect and form networks [88]. Typically free and easily accessible, these platforms support real-time communication and interaction [89,90].

The growing adoption of social media among healthcare professionals reflects a broader trend of using these tools for professional development and peer engagement [91,92]. For example, Facebook, launched in 2004 for university students, now has over one billion monthly users, and studies show that more than 90% of medical students actively use social media platforms like Facebook [87,93]. Additionally, as reported by Dua et al., educational institutions have also embraced platforms like Facebook and Twitter to enhance learning and boost student engagement [47,94,95]. With their intuitive interfaces and accessibility via smartphones, social media tools have become an integral part of medical education [21,89]. While much of the previously reported use of SNS in education was largely centred on communication and administrative tasks, emerging studies have also reported significant pedagogical benefits of SNS [96,97].

Simulation-based learning (SBL): Widely recognised as an effective pedagogical approach, SBL plays an important role in undergraduate surgical education [98]. This scoping review identified 16 studies that documented the use of SBL to enhance surgical training for medical students [99]. Traditionally, surgical education has relied on the well-known surgical maxim of 'See one, Do one, Teach one' by the American Surgeon, William Halsted, in which students observe a procedure, perform it and subsequently teach others [100]. However, concerns over patient safety and ethical considerations have rendered this approach less acceptable in recent years [101].

Kolb’s experiential learning theory emphasises that 'effective learning is the process whereby knowledge is created through the transformation of experience' [102,103]. Research has shown that active learning significantly improves skill acquisition and retention [104]. SBL aligns well with this learning model by providing learners with a safe, controlled environment to engage in all these learning phases without compromising patient care [105]. Immersive simulation is a technique that replaces or amplifies real experiences, allowing students to engage with realistic clinical scenarios without facing the inherent risks of actual practice [106-108]. By offering direct, purposeful experiences, SBL has been proven effective in bridging the gap between theoretical knowledge and clinical practice [107]. A meta-analysis revealed that technology-enhanced simulation not only improves learning outcomes but also positively influences clinician behaviours [109]. In addition to enhancing practical skills, SBL has also been shown to improve learners’ non-technical skills such as communication, teamwork and leadership [102]. One study in this review reported the utilisation of simulation in providing robotic surgery training to medical students, allowing for early robotic exposure to medical students [110,111].

The primary objective of an effective SBL is to create a sense of realism that fosters behavioural, emotional and cognitive engagement, which are essential for deep learning [107]. To achieve this, various simulation modalities have been utilised, including simulated patients, mannequins, human cadavers, task trainers, games and computer-based virtual reality simulators [109,112]. SBL can be conducted in dedicated simulation centers designed to mimic clinical settings or through in situ simulations within actual clinical environments [107]. To maximise its educational impact, it is crucial to select the appropriate simulation modality, acknowledge its limitations and provide clear pre-briefing instructions to encourage learners to approach scenarios as if they were real-life clinical situations [107].

Simulation offers students a unique opportunity to practice and make mistakes in a safe and controlled environment without jeopardising patient safety, thereby increasing their confidence and competence in performing a skill during an emergency or in a high-risk situation [108,113,114]. Another key advantage of SBL is the ability to tailor the difficulty of tasks according to the learner’s level and to pause or repeat clinical scenarios for feedback and iterative learning - options that are not feasible in real clinical settings [107]. As technology continues to advance, the role of SBL in surgical education will become increasingly significant. However, it is important to recognise that while simulation is a powerful educational tool, its effectiveness ultimately depends on the skill and strategy of the instructor facilitating the learning experience [108]. 

Game-based learning: One study in this scoping review highlighted the use of educational games to enhance patient safety learning among medical students [115]. Serious games, defined as 'video games primarily designed for educational purposes', represent an innovative and rapidly evolving approach to teaching and learning, with growing applications in surgical education [116,117]. Often referred to as 'game-based learning', 'educational games' or 'gamification' in the literature, serious games incorporate elements of play to help learners achieve specific educational objectives. Performance is typically assessed during the gaming process, allowing these tools to inform, educate and train learners in an engaging manner [118,119]. Numerous studies have demonstrated the effectiveness of serious games in the acquisition of surgical skills, establishing them as an increasingly accepted pedagogical tool in surgical education [120,121].

Virtual reality (VR): VR is an emerging technology that mimics real-world environments, providing users with a profound sense of immersion that enhances practical learning and knowledge retention [122]. Despite the growing body of literature highlighting VR’s potential in surgical education, an interesting finding from our scoping review is that only one study reported its current application in undergraduate surgical education [123,124]. This suggests that while VR holds significant promise as a tool for surgical training, its current adoption in undergraduate curricula remains limited, warranting further exploration and integration into medical education frameworks.

User experience

The majority of studies included in this review evaluated the feasibility and utility of digital tools in undergraduate surgical education by focusing on students' experiences and perceptions. Consistently, these studies found that digital technologies were well-received by students as valuable tools to supplement their learning [125,126]. A key advantage of CAL frequently highlighted by students was the ability to access course materials anytime and anywhere, allowing for greater flexibility and the opportunity to learn at their own pace, which fosters personalised learning [68,75]. This accessibility promotes a more adaptable and self-directed approach to education, aligning with modern learner preferences.

The COVID-19 pandemic accelerated the integration of digital learning into medical education, offering a unique opportunity for educators to reassess the balance between online and in-person learning, a trend likely to continue in the future [127]. Among the studies included in this review, 41 focused on e-learning in surgical education, with the majority reporting that it was well-received by students as an effective pedagogical approach [125,126,128]. Blended learning, which combines online and traditional methods, was also highly regarded by most students as an effective complement to traditional surgical teaching [83]. Similarly, the flipped classroom model, which promotes active learning and problem-solving, also received positive feedback from both students and tutors [69].

Despite these advantages, challenges with e-learning were also reported. A commonly reported challenge was time management and difficulty for students to concentrate due to distractions [22,129]. However, another study by Zhang et al. reported that over 90% of students in their study were able to maintain concentration and follow the course schedule without external supervision [125]. In addition, Boulos also found that many students missed the interpersonal engagement of face-to-face lectures, a sentiment echoed in previous studies, where students expressed feelings of isolation and a loss of motivation [22,130]. To address these issues, various teaching strategies such as team-based learning and peer teaching have been implemented to enhance engagement and sustain student interest [131].

While e-learning has been widely embraced in developed countries, its effectiveness in low- and middle-income countries is often hindered by limited internet connectivity and resources [132-135]. Although technological advancements have reduced barriers such as poor internet access, challenges like unreliable bandwidth and varying levels of digital literacy persist [126,136-139]. Previous studies have reported that enhanced technological tools have been linked to increased student satisfaction with online learning [137]. Studies in this review reported that most students primarily accessed e-learning via smartphones and laptops, which aligns with previous research identifying laptops as the predominant device for online learning [22,140]. In another study, it has been reported that messaging apps are well-received by students and the use of social media in education is associated with enhanced learning experience [21]. Messaging apps were also well-received as tools for facilitating asynchronous learning and peer collaboration, with students appreciating its ability to engage in peer discussions and collaborative learning without geographical or temporal limitations [86,96,141].

Several studies in this review examined the integration of videoconferencing in surgical education, with students generally valuing its flexibility and interactive features such as chatboxes. However, some studies, like that of Smith et al., noted concerns about limited interaction opportunities during virtual sessions [30,77,142]. Despite these gaps, other studies reported that when teleconferencing was supplemented with virtual content, learning objectives were effectively met [23]. Overall, satisfaction rates with digital learning methods were consistently higher than dissatisfaction rates, with most students preferring to continue with e-learning even after the pandemic [30,79,129,143,144].

In conclusion, most studies in this review indicated that students perceive online learning as an effective teaching pedagogy. While challenges such as concentration issues and the lack of face-to-face engagement were noted, the overall satisfaction with digital learning methods was high, with many students expressing a desire to continue using these platforms in the future.

Limitations

This study offers an important overview of the current application of digital technology in medical education to achieve surgical learning outcomes, though it is not without limitations. Firstly, the variability in study designs and methodologies among the included studies constrains the comparability of research findings. Furthermore, the results may not be generalisable to other geographical regions or educational contexts, as no standardised undergraduate surgical curriculum currently exists across medical schools. Differences in educational environments and resource availability can significantly influence the adoption and effectiveness of technological tools. Additionally, as this scoping review was conducted by a single independent reviewer, there is a potential risk of bias. Ideally, the inclusion of a second independent reviewer would have enhanced objectivity and reduced bias by incorporating multiple perspectives during data extraction and analysis.

Despite these limitations, the findings from this scoping review provide valuable insights into the current utilisation of digital technology in undergraduate medical education to meet surgical learning outcomes. These findings highlight the potential of digital tools to improve and enhance undergraduate surgical education. While acknowledging its limitations, this review offers valuable insights for educators and researchers by elucidating the roles of digital technology in enhancing and improving the delivery of undergraduate surgical education to advance teaching practices.

Recommendations for future research

A key research gap identified in this scoping review is the limited literature on the current utilisation of innovative digital technologies, such as VR, augmented reality (AR), mixed reality (MR) and 3D printing, in undergraduate surgical education. While numerous studies highlight the theoretical advantages of these advanced technologies through pilot trials, only one study within the scope of this review reported their actual implementation in undergraduate surgical curricula. This disparity suggests that although the potential benefits of these tools are well-documented, their practical application in educational settings remains largely unexamined. Addressing this gap through future research is critical to understanding how these cutting-edge innovations can be effectively integrated into undergraduate surgical education, ultimately enhancing and improving the educational experience for medical students.

## Conclusions

This scoping review sought to investigate the current landscape of digital technologies in undergraduate surgical education by systematically analysing the existing literature. A comprehensive analysis of the included studies led to the identification and synthesis of key findings, revealing three overarching themes that emerged across the body of work. First, the use of digital tools in surgical education has been steadily increasing over the past few decades, with the COVID-19 pandemic accelerating the integration of technology into surgical education. Second, this review also highlighted the key role of anatomy within surgical education, with most included studies reporting the use of digital technologies to enhance anatomy teaching. Finally, this review provided an overview of various digital tools used in surgical education and their associated user experiences. Overall, most studies indicated that digital technologies are generally well-received by students, with many advocating for their continued use in supplementing surgical education even beyond the pandemic.

This review provides a crucial foundation for understanding the evolving role of digital innovations in shaping surgical education at the undergraduate level. To enhance undergraduate surgical education, integrating appropriate digital learning tools can provide more learner-centred and personalised learning experiences. Educators must recognise that there is no ‘one-size-fits-all’ approach, and a flexible multimodal strategy is necessary to meet diverse learning needs. As technology continues to evolve and its role in education grows, this review offers valuable insights into the current use of digital tools in surgical education, highlighting opportunities for improvement and innovation to further enhance undergraduate surgical experience.
